# Is there a dose-response relationship between cannabis use and violence? A longitudinal study in individuals with severe mental disorders

**DOI:** 10.1192/j.eurpsy.2023.344

**Published:** 2023-07-19

**Authors:** M. Beaudoin, L. Dellazizzo, S. Giguere, J.-P. Guay, C.-E. Giguere, S. Potvin, A. Dumais

**Affiliations:** 1 Psychiatry and addictology, University of Montreal; 2 Research Center, Institut universitaire en sante mentale de Montreal; 3 Faculty of Medicine, McGill University; 4 Research Center, Institut national de psychiatrie légale Philippe-Pinel; 5 Criminology School, University of Montreal; 6 Centre international de criminologie comparée, Montreal, Canada

## Abstract

**Introduction:**

Recent longitudinal studies point towards the existence of a positive relationship between cannabis use and violence in people with severe mental disorders. However, the existence of a dose-response relationship between the frequency and/or the severity of cannabis use and violence has seldom been investigated.

**Objectives:**

This study aimed to determine if a dose-response relationship between cannabis use and violence exists in a psychiatric population.

**Methods:**

This observational study was conducted at the *Institut universitaire de santé mentale de Montréal* (Montréal, Canada). A total of 98 outpatients (81 males and 17 females, all over 18 years of age) with severe mental disorders were included in the analyses. Clinical evaluations were conducted every 3 months for a year. Substance use, violent behaviors, and potential covariables were assessed through self-reported assessments, urinary testing, as well as clinical, criminal, and police records. Using generalized estimating equations, the association between cannabis use frequency (non-users, occasional, regular, and frequent users, assessed using the Time-Line Follow-Back and confirmed with urinary testing) and violence was investigated, as well as the association between the severity of cannabis use (measured using the Cannabis Use Problems Identification Test – CUPIT) and violent behaviors.

**Results:**

Cannabis use frequency and severity were significant predictors of violent behaviors. After adjustment for time, age, sex, ethnicity, psychiatric diagnoses, impulsivity and use of alcohol and stimulants, odds ratios were of 1.91 (p <0.001) between each frequency profile, and 1.040 (p <0.001) for each increase of one point of the severity of cannabis use score (0 to 79).

**Image:**

**Figure:**
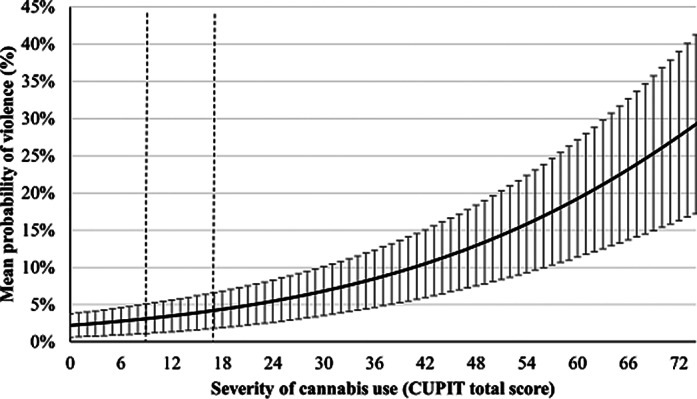

**Conclusions:**

These findings have important implications for clinicians, demonstrating that cannabis use may have serious adverse consequences in a psychiatric population. Nevertheless, the mechanisms underlying this association remain unclear.

**Disclosure of Interest:**

None Declared

